# Phage vB_PaeS-PAJD-1 Rescues Murine Mastitis Infected With Multidrug-Resistant *Pseudomonas aeruginosa*

**DOI:** 10.3389/fcimb.2021.689770

**Published:** 2021-06-11

**Authors:** Zhaofei Wang, Yibing Xue, Ya Gao, Mengting Guo, Yuanping Liu, Xinwei Zou, Yuqiang Cheng, Jingjiao Ma, Hengan Wang, Jianhe Sun, Yaxian Yan

**Affiliations:** School of Agriculture and Biology, Shanghai Jiao Tong University, Shanghai Key Laboratory of Veterinary Biotechnology, Shanghai, China

**Keywords:** *Pseudomonas aeruginosa*, MDR, phage PAJD-1, mastitis, lysin

## Abstract

*Pseudomonas aeruginosa* is a Gram-negative pathogen that causes a variety of infections in humans and animals. Due to the inappropriate use of antibiotics, multi-drug resistant (MDR) *P. aeruginosa* strains have emerged and are prevailing. In recent years, cow mastitis caused by MDR *P. aeruginosa* has attracted attention. In this study, a microbial community analysis revealed that *P. aeruginosa* could be a cause of pathogen-induced cow mastitis. Five MDR *P. aeruginosa* strains were isolated from milk diagnosed as mastitis positive. To seek an alternative antibacterial agent against MDR, *P. aeruginosa*, a lytic phage, designated vB_PaeS_PAJD-1 (PAJD-1), was isolated from dairy farm sewage. PAJD-1 was morphologically classified as *Siphoviridae* and was estimated to be about 57.9 kb. Phage PAJD-1 showed broad host ranges and a strong lytic ability. A one-step growth curve analysis showed a relatively short latency period (20 min) and a relatively high burst size (223 PFU per infected cell). Phage PAJD-1 remained stable over wide temperature and pH ranges. Intramammary-administered PAJD-1 reduced bacterial concentrations and repaired mammary glands in mice with mastitis induced by MDR *P. aeruginosa*. Furthermore, the cell wall hydrolase (termed endolysin) from phage PAJD-1 exhibited a strong bacteriolytic and a wide antibacterial spectrum against MDR *P. aeruginosa*. These findings present phage PAJD-1 as a candidate for phagotherapy against MDR *P. aeruginosa* infection.

## Introduction

Mastitis, one of the most prevalent diseases in the dairy cattle industry, leads to great economic losses for farmers caused by reduced milk production, early culling, veterinary services, and labour costs (Klaas and Zadoks, 2018). Usually, mastitis is caused by Gram-positive pathogens, such as *Staphylococcus* and *Streptococcus* (Keane, 2019). As the prevalence of udder infections due to Gram-positive pathogens is reduced to very low levels in dairy herds by the implementation of advanced mastitis control systems, the relative significance of mastitis due to Gram-negative bacterial pathogens, such as *Escherichia coli* and *P. aeruginosa*, is expected to increase (Kawai et al., 2017; Keane, 2019; El Garch et al., 2020).

*P. aeruginosa* is widely present in nature and in the intestines and skin of humans and animals (Otto, 2014; Bachta et al., 2020). In clinical treatment, the increasing resistance of *P. aeruginosa* strains to different antibiotics has led to an increase in the emergence of multi-drug resistant (MDR) *P. aeruginosa* (Javed et al., 2020; Maslova et al., 2020). Traditional antibiotics are almost ineffective against MDR *P. aeruginosa* (Tummler, 2019). In recent years, cow mastitis caused by MDR *P. aeruginosa* has attracted increasing attention and has led to significant economic losses for farmers (Kawai et al., 2017; Klaas and Zadoks, 2018). In view of this, exploring alternative treatments has tremendous value.

In the last 15 years, a marked increase in the number of identified *P. aeruginosa* bacteriophages (termed phages) has been reported, as has great progress in the phage treatment of infections caused by *P. aeruginosa* (Dzuliashvili et al., 2007; Chegini et al., 2020). Phages are the most common organism found on earth and, as such, represent great diversity in their overall host range (Khalid et al., 2020). The bacteriolysis of phages is mainly dependent on endolysin (termed lysin), which is a kind of bacterial cell wall hydrolase synthesized by phages in the late stages of infection (Wang et al., 2018; Gutierrez and Briers, 2020). Compared to traditional antibiotics, bacteriophage agents have obvious advantages, such as being simple, cheap, highly effective in killing their target bacteria and especially available in inhibiting drug-resistant bacteria, as well as causing no serious side effects (Hesse and Adhya, 2019; Principi et al., 2019). Moreover, phages are unable to infect human or animal cells because phages recognise and bind to unique bacterial receptors. Thus, the side effects associated with phage therapy in humans and animals are thought to be minimal (Rios et al., 2016).

Numerous studies have revealed that phages are able to treat various human or animal diseases caused by *P. aeruginosa*, including lung, skin, eye and other infections (Fukuda et al., 2012; Raz et al., 2019; Ng et al., 2020). However, there is no research focused on using phages to overcome mastitis caused by *P. aeruginosa*. In this study, we investigated potential pathogen diversity in cows with mastitis in Shanghai, China using microbial community analysis. A novel lytic phage, vB_PaeS_PAJD-1 (PAJD-1), was isolated from sewage in dairy farms, and its antibacterial spectrum, stability and bacteriolytic activity of its endolysin were assessed. Specifically, we evaluated the therapeutic effect of using PAJD-1 in mice with mastitis infected by MDR *P. aeruginosa*.

## Materials and Methods

### Ethics Statement

The animal experiments were carried out in accordance with animal welfare standards and approved by the Ethical Committee for Animal Experiments of Shanghai Jiao Tong University, China (Approval no. 20190103). All animal experiments complied with the guidelines of the Animal Welfare Council of China.

### Bacterial Strains and Culture Conditions

In this study, 18 clinical isolates of *P. aeruginosa* (13 hospital-acquired strains and 5 strains isolated from the milk of dairy cows with mastitis) and 2 reference strains of *P. aeruginosa* PA01 and PA14 from the American Type Culture Collection (ATCC) were used (**Table 1**). All strains were grown in Luria–Bertani broth (LB) or in a 1.5% agar medium at 37°C. Also, 5% sheep blood agar was used to isolate bacteria from milk samples. *P. aeruginosa* ATCC 27853 was used as a reference strain for identification utilising the VITEK 2 system (BioMerieux, France). Antimicrobial susceptibility testing of the isolates was performed using the Kirby–Bauer disk diffusion method as described by Fathizadeh et al. with seven antimicrobials: meropenem (MEM), ampicillin (AMP), gentamicin (GN), amikacin (AK), piperacillin/tazobactam (TZP), ciprofloxacin (CIP) and cefepime (FEP) (Fathizadeh et al., 2020). Antibiotic discs (Beijing Pronade technology co., LTD, Beijing, China) were placed on the swabbed culture and incubated for 16 to 18 h at 37°C, after which the inhibition zone was measured and each strain was determined as resistant (R)/intermediate (I)/sensitive (S) to each antibiotic tested following the instruction of the experiment provided by the manufacturer.

**Table 1:** Drug resistance of strain and lytic activity of phage PAJD-1.

No. of strains	^a^Source	^b^EOP	Antibiotic susceptibility						
			MEM	AMP	GN	AK	TZP	CIP	FEP
**PA01**	I	-^c^	S	R	S	S	S	S	S
**PA14**	I	1	S	R	I	S	S	S	I
**PAmas1**	II	0.28	S	R	I	I	S	S	R
**PAmas2**	II	0.26	S	R	S	S	S	S	S
**PAmas3**	II	0.44	I	R	I	S	S	S	S
**PAmas4**	II	0.27	S	R	S	S	S	S	S
**PAmas5**	II	0.84	S	R	S	I	S	S	S
**PA8094**	III	–	S	R	I	S	S	S	I
**PA8299**	III	0.32	S	R	S	S	R	S	R
**PA8243**	III	–	S	R	S	S	S	S	S
**PA7959**	III	0.41 × 10^-3^	S	R	S	S	S	S	I
**PA8070**	III	0.68 × 10^-2^	S	S	I	S	S	R	I
**PA7978**	III	0.38	S	R	I	I	S	S	S
**PA8244**	III	0.64	S	R	S	S	S	S	S
**PA8408**	III	0.21 × 10^-3^	S	R	S	S	R	S	R
**PA8362**	III	0.71	S	R	R	S	R	R	R
**PA7979**	III	0.91	S	R	S	S	S	S	I
**PA8177**	III	–	S	R	S	S	S	S	S
**PA8333**	III	0.21	S	R	S	S	S	S	I
**PA8218**	III	0.27	S	R	S	S	R	S	R

a I, purchased from American Type Culture Collection; II, clinically-isolated strains from the milk of dairy cows with mastitis; III, hospital-acquired strains.

b EOP, efficiency of plating (EOP = phage titre on test bacterium/phage titre on strains PA14). Assays were conducted at least three times. The data shown are means from three independent experiments.

c no plaque on target bacterium.

R, resistant; I, intermediate; S, sensitive.

### Sample Analysis of Microbial Community Composition and Diversity From a Dairy Farm

The udder surfaces of cows with mastitis from three dairy farms (at least three samples per dairy farm) in Shanghai were sampled using sterile cotton swabs. These samples were then suspended in sterilised phosphate-buffered saline (PBS) buffer and placed in sterilised, RNase-free tubes. After homogeneous mixing, centrifugation, and filtration, 2 mL of the filtrate was stored at -80°C. The total microbial RNA samples were extracted using an RNA Mini Kit (Bio-Rad, Hercules, CA, USA). RNA samples were reverse-transcribed to complementary DNA (cDNA) using the iScript cDNA Synthesis Kit (Bio-Rad). The quantity and quality of the extracted cDNA were measured using a NanoDrop ND-1000 spectrophotometer (Thermo, Waltham, MA, USA) and agarose gel electrophoresis, respectively. 16S rRNA gene amplicon sequencing and analysis were performed as described previously (Morella et al., 2018). The cDNA was amplified using the primer sets 515F and 806R, which targeted the V4 region of the bacterial 16S rDNA, with the reverse primer containing a 6-bp error-correcting barcode unique to each sample (Caporaso et al., 2012). Sequencing by synthesis was performed on an Illumina HiSeq MiSeq platform (Shanghai Personal Biotechnology Co., Ltd., Shanghai, China).

The Quantitative Insights into Microbial Ecology (QIIME, v1.8.0) pipeline was employed to process the sequencing data, as previously described (Caporaso et al., 2010). Briefly, the high-quality sequences were clustered into operational taxonomic units (OTUs) at 97% sequence identity by UCLUST (Edgar, 2010). A representative sequence was selected from each OTU using default parameters. The OTU taxonomic classification was conducted by BLAST, searching the representative sequences set against the Greengenes Database (Desantis et al., 2006) using the best hit (Altschul et al., 1997). An OTU table was further generated to record the abundance of each OTU in each sample and the taxonomy of these OTUs. OTUs containing less than 0.001% of the total sequences across all samples were discarded. To minimize the difference in sequencing depth across samples, an averaged, rounded, rarefied OTU table was generated by averaging 100 evenly resampled OTU subsets under 90% of the minimum sequencing depth for further analysis. Taxonomy assignment of OTUs was performed by comparing sequences to the Greengenes database. The Mann–Whitney *U* test was used to test for the significance of alpha diversity. A two-sided Student’s *t-*test was conducted to determine the significance of beta diversity between sample groups. Linear discriminant analysis coupled with effect size (LEfSe) was performed to identify the bacterial taxa represented between groups at the genus or higher taxonomic levels (Segata et al., 2011).

### Phage Isolation, Purification and Host Range Determination

The isolation method described by Wang et al. was applied for the isolation of *P. aeruginosa* phages, with some modifications (Wang et al., 2016). Sewage from dairy farms was centrifuged at 5,000 × *g* (centrifuging radius = 17.6 cm) for 20 min at 4°C. The supernatants were passed through 0.22-μm pore size membrane filters. *P. aeruginosa* PA14 of the logarithmic phase was cultured overnight together with sewage samples in LB broth at 37°C with shaking at 180 rpm. The culture was centrifuged at 5,000 × *g* for 20 min at 4°C; the supernatant was then filtered through 0.22-μm pore size membrane filters and checked for the presence of lytic phages by *P. aeruginosa* PA14 using the double-layer agar plate method as previously described (Wang et al., 2016). After overnight incubation, the formation of obvious zones suggested the presence of a lytic phage, which was purified by three rounds of single-plaque isolation.

For purification, single-phage plaques were precipitated in the presence of 10% (wt/vol) polyethylene glycol (PEG) 8000 and 1 M NaCl at 4°C for at least 1 h. The precipitate was collected by centrifugation at 10,000 × *g* for 10 min at 4°C and suspended in SM buffer (100 mM NaCl, 10 mM MgSO_4_·7H_2_O and 50 mM Tris·HCl pH 7.5). After the addition of 0.5 g/mL CsCl, the mixture was layered on top of CsCl step gradients (densities of 1.15, 1.45, 1.50 and 1.70 g/mL) in Ultra-Clear centrifugation tubes and centrifuged at 28,000 × *g* for 2 h at 4°C, dialysed in SM buffer. Phages were stored at 4°C for further experiments.

The PAJD-1 phage was screened against *S. aureus* strains using the efficiency of the plating method (EOP = phage titre on test bacterium/phage titre on strains PA14) to determine the effectiveness and host range against a variety of target bacteria. Ten-fold serial dilutions of phage suspensions (100 μL) were mixed with 100 μL of bacteria (1 × 10^8^ CFU/mL), incubated for 5 min at room temperature (25°C) and plated as double layers on LB to determine phage titres.

### Transmission Electron Microscopy (TEM) of Phage Particles

The purified phage was loaded onto a copper grid for 10 min, negatively stained with 2% (v/v) phosphotungstic acid (pH 6.7) and dried. The morphology of the phage was observed using a FEI TEM Tecnai G2 Spirit Biotwin (FEI, Hillsboro, US) at an accelerating voltage of 120 kV.

### Genome Sequencing and Annotation

Purified PAJD-1 phage genomic DNA was prepared using phenol–chloroform extraction and ethanol precipitation methods, as described previously (Wang et al., 2016). The Illumina MiSeq system was used for the PAJD-1 phage whole genome analysis. Sequence alignments were carried out using the Accelrys DS Gene software package of Accelrys Inc. (USA). Putative open reading frames were suggested using the algorithms of the software packages Accelrys Gene v2.5 (Accelrys Inc.) and ORF Finder (NCBI). Identity values were calculated using different BLAST algorithms (http://www.ncbi.nlm.nih.gov/BLAST/) on the NCBI homepage. The sequence of the PAJD-1 phage was submitted to the NCBI (GenBank accession number: MW835180).

### One-Step Growth Analysis of Phages

To determine the one-step growth of PAJD-1, *P. aeruginosa* PA14 was used as the indicator strain. One-step growth experiments were performed with a modification to the methods described previously (Pajunen et al., 2000). Briefly, PAJD-1 phage (1 × 10^6^ PFU) was added at a MOI of 0.1 to the cells of *P. aeruginosa* (1 × 10^7^ CFU) and allowed to adsorb for 10 min at 37°C. The mixture was then centrifuged at 4°C for 1 min at a speed of 12,000 × *g*. After the supernatants were removed, the pellets containing the phage-infected bacterial cells were suspended in fresh LB and incubated with shaking at 180 rpm and 37°C. Partial samples were taken at 10 min intervals, and the titrations from the aliquots were immediately determined using the double-layer agar plate method. Burst size was calculated as the ratio between the number of total released phages and the number of infected bacterial cells. This assay was performed in triplicate.

### Adsorption Analyses of Phages

The adsorption rate of PAJD-1 was performed as previously described, with some modifications (Ong et al., 2020). Briefly, *P. aeruginosa* PA14 (1 × 10^7^ CFU/mL) was mixed with phage (1 × 10^6^ PFU/mL) incubated at 37°C. Samples of the mixture (100 μL) were taken at 5, 10, 15, and 20 min, and filtered (0.22-μm pore size membrane) immediately. The filtered supernatants (unadsorbed phages) were determined using the double-layer agar plate method. The adsorption rate of PAJD-1 (%) = (1 unadsorbed phages/initial concentration of phage) × 100%.

### Phage Stability Assay

To determine phage stability at different temperatures (25°C, 37°C, 45°C, 50°C, 55°C, 60°C, 65°C and 70°C), an aliquot of the PAJD-1 phage was taken after 1 h of incubation, and the titres of the phage were assayed using the double-layer agar plate method. To determine the optimum storage temperature of the PAJD-1, phages were stored at 4°C, -20°C and -80°C for 6 months, after which their bactericidal activity (titres of the phage) were determined by the double-layer agar plate method and compared with initial titres. To test for phage stability at the different pH values, titres were determined after the phage lysates were diluted (1:100) in SM buffer at different pH values and kept at 37°C for 3 h using the double-layer agar plate method.

### Phage Bacteriolytic Assay *In Vivo*

To improve the safety of the phage used in animals, an affinity matrix of modified polymyxin B (PMB) (GenScript, Piscataway, Nanjing, China) was used to remove phage endotoxins. Furthermore, the endotoxin levels of the phage were evaluated by the colorimetric method following the recommendations of the manufacturer (GenScript). The end-product was measured spectrophotometrically in a microplate reader. Female lactating BALB/c specific-pathogen-free (SPF) mice (10–14 days after the birth of their offspring) were purchased from the Experimental Animal Center, Shanghai Jiao Tong University. A mixture of ketamine 100 mg/kg (Imalgene, Merial Laboratorios, S.A) and xylacine 10 mg/kg (Rompun, Bayer Health Care) were administered intraperitoneally as anaesthesia for the mice. A syringe with a 33-gauge blunt-end needle was used to inoculate both the L4 (on the left) and R4 (on the right) of the fourth abdominal mammary gland pair with 1 × 10^5^ CFU/gland (50 μL) of *P. aeruginosa* PAmas5. After 6 h, the phage-treated groups (*n* = 5) received an intramammary dose of 1 × 10^6^ PFU/gland (50 μL). After 6 h, PAmas5-infected mice were treated through intramammary injection of 20 μg/gland ceftiofur sodium (50 μL) and 50 μL/gland PBS as the antibiotic-treated (*n* = 5) and PBS control (*n* = 5) groups, respectively. After a 24-h period, the mammary glands of the mice were photographed. The L4 mammary glands were aseptically removed, individually weighed, serially diluted in PBS (1:9) and plated in agar containing ampicillin (50 μg/mL) to determine the number of CFU/gland. The R4 mammary glands were gently removed and immediately placed in 4% formalin. Formalin-fixed tissues were processed and stained with hematoxylin and eosin (H&E) and toluidine blue using a routine staining procedure and were subsequently analysed using microscopy. (Note that the mammary glands removed from healthy lactating mice served as positive controls for histopathology).

The alteration of mammary gland histology was measured semi-quantitatively as described previously with slight modifications (Camperio et al., 2017). A scale from 0 to 3 was applied to the following alterations: 0 = normal healthy lactating alveoli without pathological changes; 1 = a minimal degree of necrotic acinar epithelial cells and/or interstitial inflammation and relatively normal mammary glands; 2 = a moderate degree of necrotic acinar epithelial cells and/or interstitial inflammation and relatively normal mammary glands; 3 = severe tissue damage, a very large number of interstitial inflammatory cells and extensive necrotic areas. All slides were assessed by three blinded observers (ZW, MG and YY) using a light microscope, and the discordant cases were reviewed at a multi-head microscope until a consensus was reached.

### Cloning, Expression and Purification of the Lysin of the PAJD-1 Phage (PlyPAJD-1)

The PlyPAJD-1-encoding region was PCR-amplified with primers 5’ *Bam*HI_JDlys (CGCGGATCCATGAACGGTGCGACATAC, where the *Bam*HI site is underlined) and 3’ *Xho*I_JDlys (CCGCTCGAGTTATCGCCAATCCACTTTCTT, where the *Xho*I site is underlined), using the genomic DNA of the PAJD-1 phage as a template for PlyPAJD-1. The PCR product was digested with *Bam*HI/*Xho*I and cloned into the pET-28a vector. Constituted plasmids were expressed in *E. coli* BL21 (DE3) grown to an optical density of 0.6 at 600 nm (OD_600_) at 37°C, induced with 1 mM isopropyl-β-D-thiogalactoside (IPTG) and expressed for 14 h at 16°C. Cells were disrupted by sonication and purified with a Ni Sepharose 6 Fast Flow resin gravity column (GE Healthcare BioSciences, Pittsburgh, USA), as described previously (Zhang et al., 2016).

### Bactericidal Activity and Lytic Spectrum of PlyPAJD-1

To determine the bactericidal activity of PlyPAJD-1 against *P. aeruginosa* PA14, 20 mL of early log-phase bacteria cells (5 × 10^8^ CFU/mL) were pelleted and resuspended in 20 mM Tris–HCl buffer (pH 7.5) supplemented with 0.1 M EDTA for 5 min at room temperature. Then, cells were pelleted and thrice washed with PBS to remove the remaining EDTA. Next, the washed cells were mixed with 10 mL semisolid TSB medium at 42°C and then spotted on a plate. After solidification, 100 μL of PlyPAJD-1 protein (1mg/mL) was put into a punched hole. PBS was spotted into the other hole as a negative control. The plates were incubated for 12 h at 37°C, and the inhibition zone was used to check the lytic activity of PlyPAJD-1. The assay was performed at least three times in biological repeats.

To verify the lytic spectrum of PlyPAJD-1, a 96-well plate was used, as described by Wang et al. with minor modifications (Wang et al., 2018). Briefly, fresh bacterial cells (1×10^9^ CFU/mL) were pelleted and resuspended in 20 mM Tris–HCl buffer (pH 7.5) supplemented with 0.1 M EDTA for 5 min at room temperature. Then, cells (5×10^8^ CFU/mL) were pelleted and thrice washed with PBS to remove the remaining EDTA. The lytic effect was monitored by blending 100 μL of bacterial suspension with 100 μL (1 mg/mL) of PlyPAJD-1 in a 96-well microtiter plate. The OD_600_ values were monitored after 2h. The decrease in bacterial turbidity was calculated by the OD_600_ after 2 h/original OD_600_ of the mixture. The assay was performed at least three times in biological repeats.

### Statistical Analysis

In all experiments, the data were plotted using GraphPad Prism 6.01 (GraphPad Software, Inc., San Diego, CA, USA). The statistical significance of changes between groups was assessed with an unpaired Student’s *t*-test. The *p*-values are indicated in the figure legends.

## Results

### *P. aeruginosa* Could Cause Pathogen-Induced Cow Mastitis

**Figure 1:**
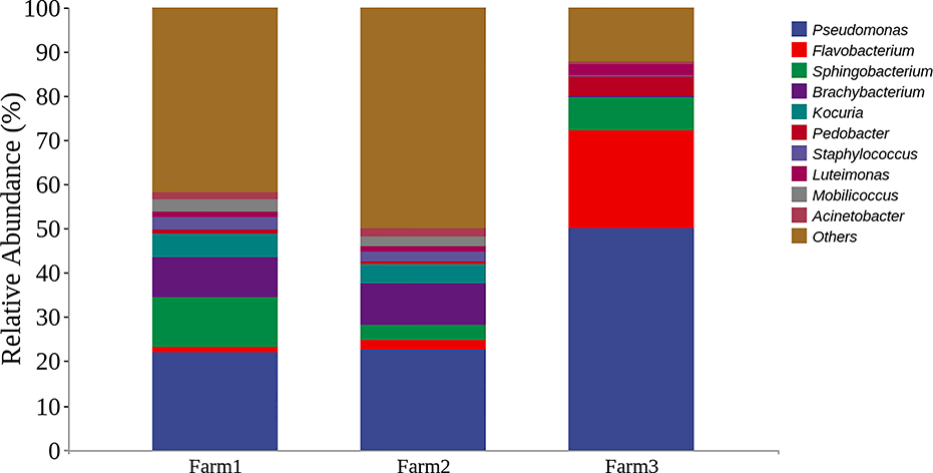
Representation of the top bacterial genus in cows with mastitis by microbial community analysis. Bar graphs show the relative abundance of the top 10 bacterial genera from three dairy farms in Shanghai, China.

To evaluate the microbes from three dairy farms in Shanghai, microbial community analysis of the udder surface of cows with mastitis was carried out, as shown in **Figure 1**. The results revealed that the predominant genera for cows with mastitis mainly included *Pseudomonas* (22.12%, 22.71% and 50.16%), *Flavobacterium* (1.15%, 2.21% and 22.06%), *Brachybacterium* (8.97%, 9.46% and 0.075%), and *Staphylococcus* (2.97%, 2.16% and 0.36%). Furthermore, five *P. aeruginosa* strains identified by the VITEK 2 system were isolated from milk obtained from cows diagnosed with mastitis (**Tables S1**–**S5**). In particular, these strains were multidrug resistant (**Table 1**). These findings suggest that *Pseudomonas*, especially *P. aeruginosa*, could be a potential pathogen in dairy farms in Shanghai.

### Isolation, Identification and Host Range Determination of *Pseudomonas* Phage PAJD-1

**Figure 2:**
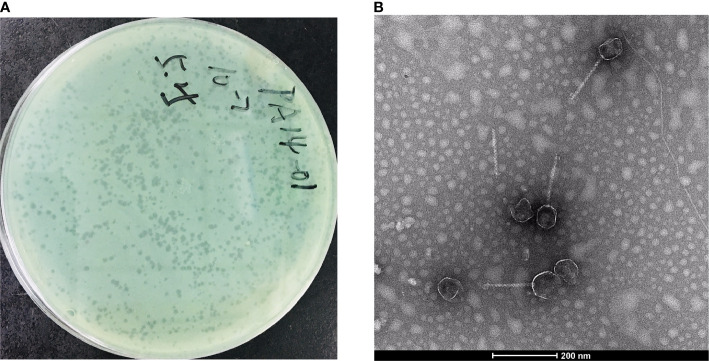
Morphological images of PAJD-1. **(A)** Single-plaque in a double-layer agar plate. **(B)** Transmission electron microscopy image of PAJD-1.

In this study, we isolated a lytic *Pseudomonas* phage, PAJD-1, from faecal sewage in dairy farms in Shanghai, China. Using *P. aeruginosa* PA14 as the host strain, the phage formed plaques 1 to 2 mm in diameter (**Figure 2A**). Among 20 P*. aeruginosa* strains, 80% (16/20) of the isolates were lysed by PAJD-1. In particular, PAJD-1 could effectively lyse five MDR *P. aeruginosa* strains isolated from dairy farms (**Table 1**).

The morphology of the isolated phage PAJD-1 was further characterised. TEM showed that the PAJD-1 particle had an isometric head of approximately 50 nm and a long, noncontractile tail with a length of approximately 200 nm (**Figure 2B**). Thus, it was morphologically similar to phages of the family *Siphoviridae* according to the classification of the International Committee on Taxonomy of Viruses (ICTV).

### General Features of the PAJD-1 Genome

**Figure 3:**
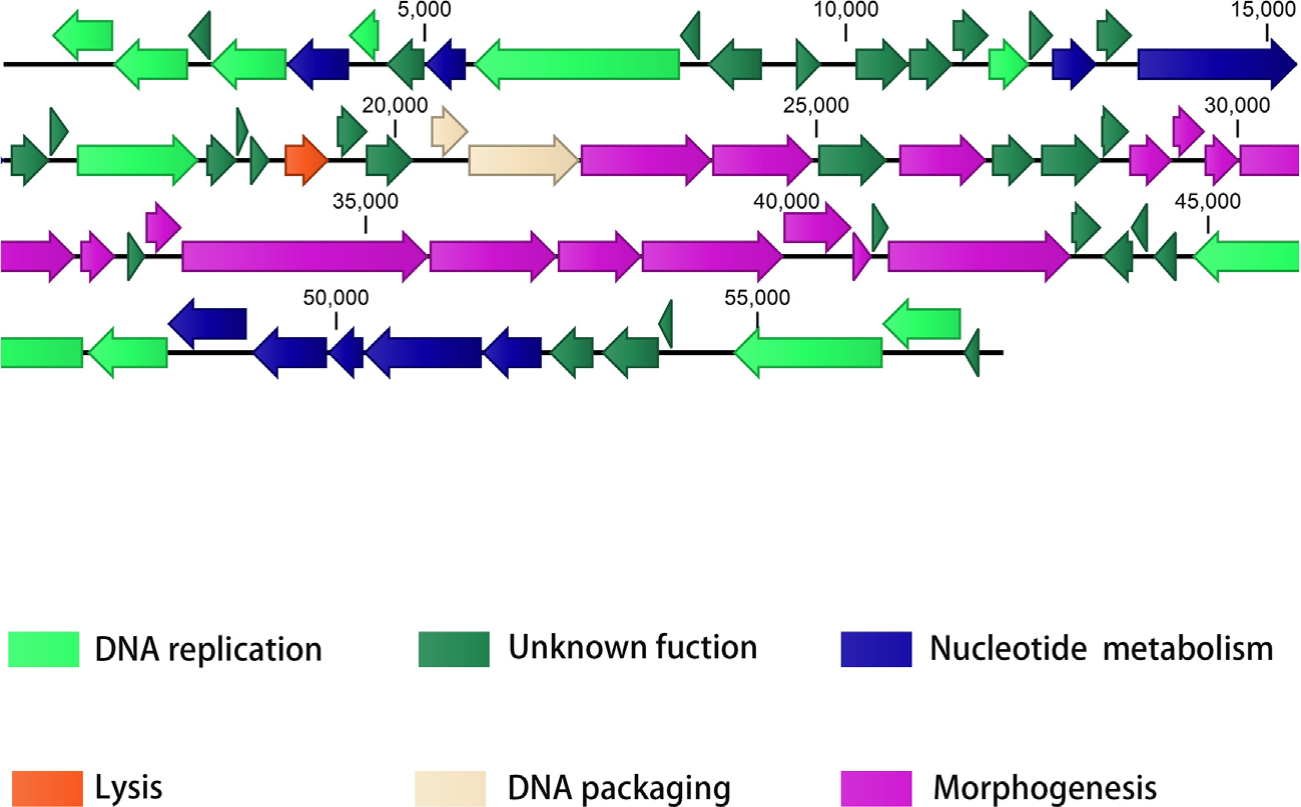
The genome map of PAJD-1. The ORFs in the direction of transcription is shown by arrows. Groups of functional genes are indicated by different colours, including morphogenesis (purple), DNA replication (light green), nucleotide metabolism (blue), lysis (red), DNA packaging (pink), and hypothetical proteins (bottle green) modules.

The PAJD-1 genome comprised 57.9 kb, double-stranded DNA and an average G+C content of 58.32%. Analysis *via* BLAST showed that the genome sequence of PAJD-1 belonged to a NP1-like phage (**Table 2**), which showed partial homology to phage NP1 (94%), phage Quinobequin (93%), phage PaMx25 (93%), phage PaMx25 (93%), phage PaMx25 (93%) and phage PaMx25 (93%). As shown in the whole-genome arrangement map (**Figure 3**), 72 open reading frames (ORFs) were defined as potential genes of PAJD-1. The genes of PAJD-1 were categorized into six modules: morphogenesis (purple), such as head or tail structural proteins and some putative virion synthetic proteins; DNA replication (light green), such as DNA topoisomerase, DNA ligase and ribonuclease; nucleotide metabolism (blue), such as thymidylate synthase, ribonucleotide reductase glutamine amidotransferases and GTP cyclohydrolase; lysis modules (lysozyme-like transglycosylase, red); DNA packaging (pink), including terminase large subunit and terminase small subunit; and hypothetical proteins (bottle green). Among these ORFs, a putative tail structural protein (ORF 48) had the lowest homology (less than 65%) with the related genes of the above-mentioned phages, which are homologous to the genome of bacteriophage PAJD-1 (**Table 3**). Furthermore, BLAST analysis identified no ORFs associated with drug resistance, pathogenicity or lysogenisation, such as site-specific integrases or repressors in the whole-genome of PAJD-1.

**Table 2:** The sequence identity of the PAJD-1 genome with other *Pseudomonas* phage.

Accession	Other phages	Phage type	Genome size (bp)	Morphology	Query cover
**KX129925.1**	*Pseudomonas* phage NP1	Lytic	58566	Siphoviridae	94%
**MN504636.1**	*Pseudomonas* phage Quinobequin-P09	Lytic	58277	Siphoviridae	93%
**NC_041953.1**	*Pseudomonas* phage PaMx25	Lytic	57899	Siphoviridae	93%
**KX898399.1**	*Pseudomonas* phage JG012	Lytic	58359	Siphoviridae	93%
**KX898400.1**	*Pseudomonas* phage JG054	Lytic	57839	Siphoviridae	90%

**Table 3:** The sequence identity of the PAJD-1 ORF48 with other *Pseudomonas* phage.

Accession	Description	The corresponding phage	Identity
**KX129925.1**	putative structural protein	*Pseudomonas* phage JG012	64.3%
**MN504636.1**	Putative virion structural protein	*Pseudomonas* phage NP1	63.51%
**NC_041953.1**	hypothetical protein	*Pseudomonas* phage Quinobequin-P09	61.86%
**KX898399.1**	hypothetical protein	*Pseudomonas* phage JG054	60.83%
**KX898400.1**	structural protein	*Pseudomonas* phage PaMx25	60.43%
**YP_006561077.1**	tail fiber structural protein	*Pseudomonas* phage MP1412	52.38%

### Determination of the One-Step Growth Curve and Adsorption Ability of PAJD-1

**Figure 4:**
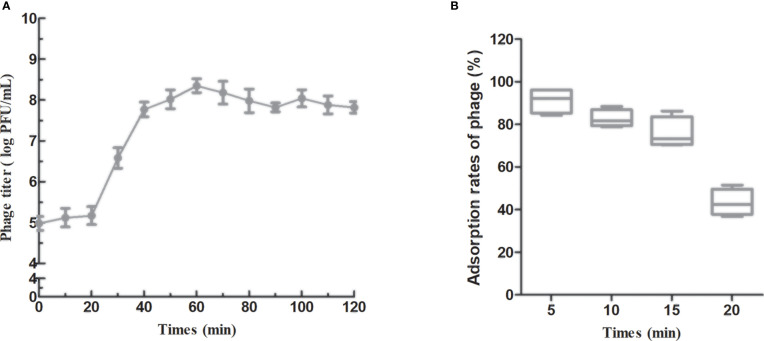
*In vitro* characterization of phage PAJD-1. **(A)** One-step growth curves and **(B)** adsorption rates of PAJD-1. Results are shown as means ± SEM from triplicate experiments.

To identify the different phases of the phage infection process, a one-step growth curve of PAJD-1 was determined. The results revealed that a latent period (defined as the time interval between the absorption and the beginning of the first burst) was about 20 min, and the burst size was estimated as 223 PFU per infected cell (**Figure 4A**), which was calculated as the ratio of the final count of liberated phage particles to the initial count of infected bacterial cells. Furthermore, the adsorption rates of PAJD-1 were determined. After 5 min of phage–bacteria incubation, about 95% of the phage particles were attached to the host cells (**Figure 4B**). After 30 min, only 65% of the phage particles were adsorbed, indicating that phage PAJD-1 had begun to lyse bacteria and that progeny phages were produced (**Figure 4B**).

### The Temperature and pH Stability of Phage PAJD-1

**Figure 5:**
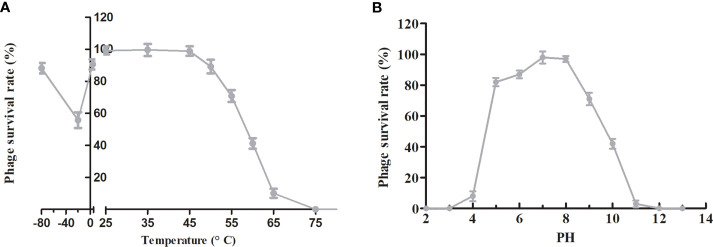
Stability tests of phage PAJD-1. **(A)** Temperature stability: phage PAJD-1 was incubated at various temperatures as indicated. **(B)** pH stability: phage PAJD-1 was incubated at different pH conditions for 3 h. Results are shown as means ± SEM from triplicate experiments.

To evaluate the suitability of phage PAJD-1 for potential clinical application in the future, a series of physical and chemical stabilities of phage PAJD-1 were examined. The stability of phage PAJD-1 was investigated at several temperatures. We found that the activity of phage PAJD-1 remained stable over a wide range of temperatures up to 50°C. Higher temperatures resulted in progressive inactivation. Phage PAJD-1 was completely inactivated when heated to 65°C (**Figure 5A**). Moreover, **Figure 5A** shows that PAJD-1 maintained more than 80% of its bactericidal activity when stored at 4°C and -80°C for 6 months. pH stability was evaluated in SM buffers adjusted to values between pH 2 and pH 12. Phage PAJD-1 remained at a relatively high survival rate (more than 80%) in the pH range of 5 to 8. Beyond these values, the activity decreased dramatically (**Figure 5B**).

### Phage Treatment in a Mouse Model of *P. aeruginosa*-Induced Mastitis

**Figure 6:**
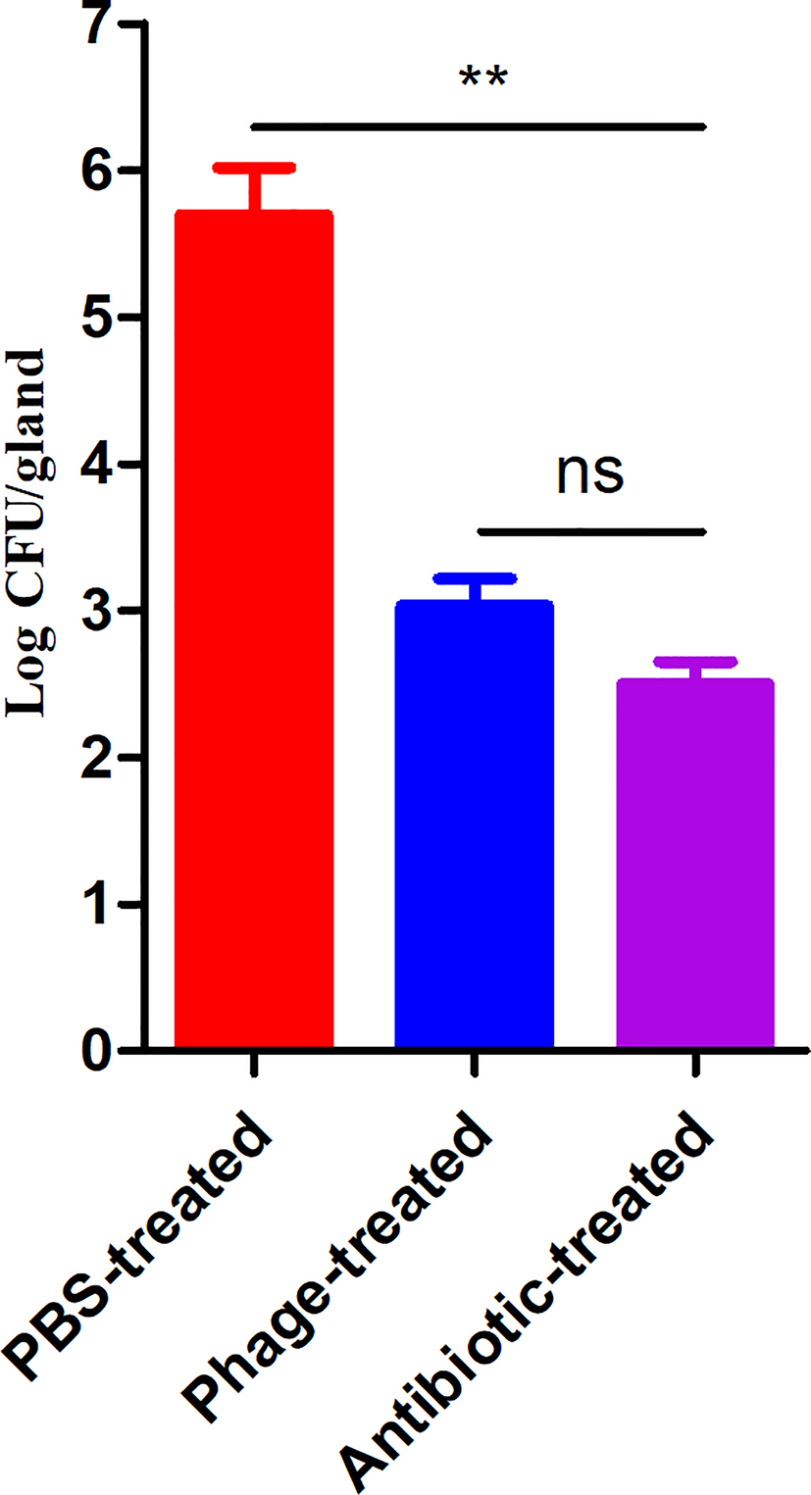
*P. aeruginosa* concentrations in the mammary glands of mice treated with phage PAJD-1. The fourth abdominal mammary gland was infected with *P. aeruginosa* PAmas5. After 6 h, the mice were treated with the phage, an antibiotic and PBS. After 24 h, the L4 mammary glands were aseptically removed. Results are shown as means ± SEM from triplicate experiments. Significant differences (*p* < 0.01) are indicated by asterisks and ns represents no significant.

To evaluate the therapeutic potential of phage PAJD-1 *in vivo*, assays were performed on female lactating mice infected with MDR *P. aeruginosa* strain PAmas5, which was isolated from milk samples diagnosed as mastitis positive and efficiently lysed by PAJD-1 *in vitro* (**Table 1**). The results showed that the mammary glands of mice treated with PBS had the highest CFU burden (about log 5.79 CFU/gland). By contrast, mammary glands from mice treated with antibiotic had the lowest CFU burden (about log 2.41 CFU/gland). Mammary glands from the phage-treated mice had median CFU burdens of about log 3.08 CFU/gland, significantly lower than the PBS control group (*p* < 0.01, **Figure 6**).

**Figure 7:**
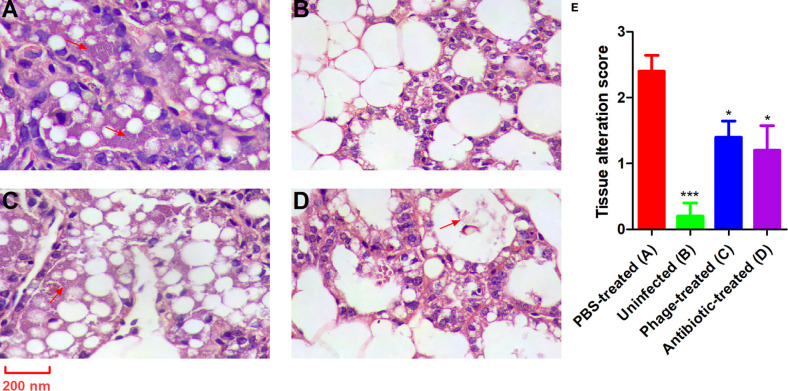
PAJD-1 reduced *P. aeruginosa*-induced mammary gland lesions of mice. Mice were infected with *P. aeruginosa* PAmas5 strains and treated with **(C)** PAJD-1 and **(D)** ceftiofur sodium. **(A)** Mice were treated with PBS after infection as a medium-treated group. **(B)** An uninfected mouse served as a positive control. **(E)** The tissue alteration score was measured in tissue sections above. The scoring criteria were present in the materials and methods section. Mammary glands were collected 24 h after treatment and processed for H&E staining and microscopic examination. Results are shown as means ± SEM. Asterisks indicate when the tissue alteration score of uninfected mice and mice treated with PAJD-1 or ceftiofur sodium after infection were significantly lower (***p < 0.001 and *p < 0.05) than untreated mice after infection. The red arrows indicate neutrophilic infiltration. Magnification × 100, scale bars represent 200 nm.

Damage to the mammary glands was observed by anatomical photograph in the mice (**Figure S1**). Representative images after histopathological examination are shown in **Figure 7**. The histopathological changes were semi-quantified as the tissue alteration score as shown in **Figure 7E**. Healthy lactating mice revealed normal healthy lactating alveoli without pathological changes (**Figure 7B**); however, the mammary glands of the PBS-treated mice with *P. aeruginosa*-induced mastitis showed obvious oedema and bleeding (**Figure S1A**). The H&E results showed an obvious intraglandular neutrophilic infiltration. The acinar epithelial cells were necrotic and detached with the acinar space infiltrated by a very large number of interstitial inflammatory cells (**Figure 7A**). By contrast, the mice treated with antibiotics exhibited relatively normal mammary glands (slight hyperaemia), and a minimal degree of necrotic acinar epithelial cells and interstitial inflammation were identified (**Figures S1D** and **7D**). Compared with the PBS-treated mice, the mammary gland tissues of the mice from the phage-treated group showed relatively moderate oedema and bleeding (**Figure S1C**). Patchy, minimal neutrophilic inflammation and necrotic acinar epithelial cells were observed only in several glands (**Figure 7C**).

### The Expression, Purification and Lytic Activity of PlyPAJD-1

**Figure 8:**
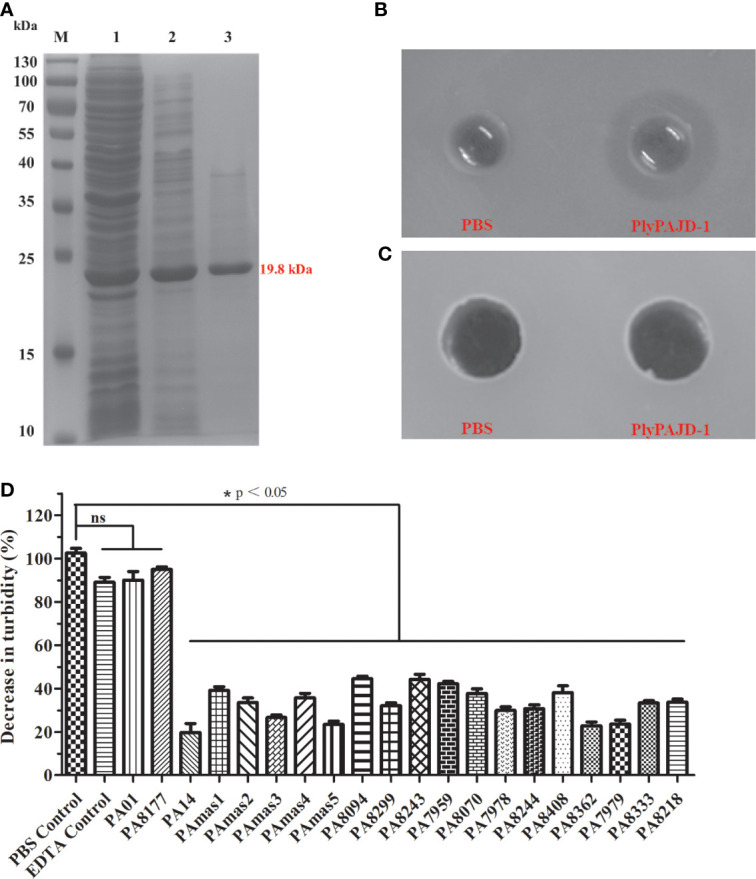
The expression and lytic activity of PlyPAJD-1. **(A)** Sodium dodecyl sulphate–polyacrylamide gel electrophoresis (SDS-PAGE) analysis of PlyPAJD-1. M, molecular size marker; Lane 1, unpurified protein; Lane 2, flow-through sample; Lane 3, purified PlyPAJD-1 (19.8 kDa); **(B, C)** show the inhibition zone around the punched hole where the PlyPAJD-1 and PBS in bacteria pre-treated **(B)** with EDTA or **(C)** without EDTA, respectively. **(D)** The lytic spectrum of PlyPAJD-1 against different *P. aeruginosa.* Results are shown as means ± SEM from triplicate experiments. Significant differences (*p* < 0.05) are indicated by asterisks and ns represents no significant.

To determine the bacteriolytic activity of the lysin (PlyPAJD-1) from phage PAJD-1, PlyPAJD-1 was expressed and purified. The results showed that PlyPAJD-1 was successfully expressed in *E. coli* BL-21, and the size was 19.5 kDa (**Figure 8A**). The purified protein concentration was 2.1 mg/mL (data not shown). To evaluate the bacteriolytic activity of the PlyPAJD-1 protein against *P. aeruginosa*, a plate lytic assay was performed. The results showed a marked inhibition zone around the punched hole where the PlyPAJD-1 lay (**Figure 8B**). Notably, PlyPAJD-1 could not kill *P. aeruginosa* directly without pre-treatment with EDTA (**Figure 8C**). The lytic spectrum of PlyPAJD-1 against different *P. aeruginosa* is shown in ​**Figure 8D**. The result showed that after treatment with PlyPAJD-1, the concentrations of 90% (18/20) of the *P. aeruginosa* strains were significantly reduced compared with the strains treated with PBS.

## Discussion

In recent years, the incidence of clinical mastitis due to *P. aeruginosa* and the associated risk of large economic losses have increased in large dairy herds, causing significant problems for affected farmers (Kawai et al., 2017; Klaas and Zadoks, 2018). Consistent with the previous issue, our study found that *P. aeruginosa* might become the major pathogenic bacterium that survives on the milk or udder surface of cows with mastitis (Kawai et al., 2017; Schauer et al., 2021). Notably, all five *P. aeruginosa* isolates from these cows were MDR. Therefore, there is an urgent need for novel therapies to treat and prevent bovine mastitis caused by *P. aeruginosa*.

With the rapid development of phage therapy, the use of phages against *P. aeruginosa* infections has been widely studied in experimental infections in humans and animals (Raz et al., 2019; Ng et al., 2020). In this study, we successfully isolated a lytic phage against *P. aeruginosa* from sewage in a dairy farm. The isolated PAJD-1, which was different from other *Siphoviridae* of previous reports (Amgarten et al., 2017; Jeon and Yong, 2019), showed intraspecific broad-spectrum lytic activities and good bactericidal activity against MDR *P. aeruginosa* strains (**Table 1**). It is known that the adsorption capacity, which is affected by the receptor-binding proteins (RBPs) of a phage, is the most important factor in determining its bactericidal broad-spectrum (Rios et al., 2016). Studies have found that through the exchange or insertion of RBPs (for instance, tail fibre protein) of virulent phages, such as members of the T3 or T7 families, the host range of related phages has been modified and expanded (Ando et al., 2015; Yehl et al., 2019). In this study, the hypothetical tail fibre protein of PAJD-1 (gb48) showed relatively low homology to other NP1-like phages (less than 65%), suggesting that phage PAJD-1 might have a different or relatively broad lytic spectrum than other NP1-like phages. Besides a wide host range, an optimal phage therapeutic agent necessitates certain features, such as a strictly lytic lifestyle, no toxins, and antibiotic-resistant genes (Rios et al., 2016). In addition to the intraspecific broad spectrum, the clarity of the genetic background is also a key factor for the application of bacteriophages (Rios et al., 2016). Genome sequence analysis showed that no ORFs associated with drug resistance, pathogenicity, or lysogenisations (such as site-specific integrases or repressors) were identified, which indicated that PAJD-1 has the potential for biocontrol and therapy.

Moreover, a high number of therapeutic phages (at least 1 × 10^8^ PFU/mL) must be used to ensure sufficient contact and rapid infection of targeted cells. Selected phages should be easily propagated in liquid media with high titre (Brovko et al., 2012). Our study found that phage PAJD-1 rapidly proliferated (from 1 × 10^5^ PFU/mL to 3 × 10^8^ PFU/mL) within *P. aeruginosa* at 60 min after infection based on assays of one-step kinetics. Moreover, PAJD-1 showed a relatively short latent adsorption period (20 min) but a remarkable adsorption capacity (95% of adsorption rate). These findings indicate that the concentration of PAJD-1 can be achieved quickly and efficiently for application.

Among the physiological properties of phages, temperature and pH stability are considered important factors in the survival of phages during infectivity and storage (Wang et al., 2016). Therefore, phages that have high stability at various temperatures and pH values are better candidates for applications, such as alternative therapeutic agents (Rios et al., 2016). Our study found that phage PAJD-1 showed a relatively broad range of temperature tolerance and pH stability. PAJD-1 stored at 4°C and -80°C for at least half a year maintained antibacterial activity. These data can be used to optimize the storage and therapeutic application of phages under various physicochemical conditions.

Scholars have reported many successful outcomes for the local and systemic application of phages in the treatment of human and animal infections with *P. aeruginosa*. For example, Jeon and Yong reported that nasal inhalation of phage significantly decreased the *P. aeruginosa* concentrations in the lungs of mice with pneumonia (Jeon and Yong, 2019). Moreover, there have been two clinical trials of *P. aeruginosa* phage therapy: one trial involving treatment of a *P. aeruginosa*-infected ear (Wright et al., 2009) and the other a treatment of burn infections (Jault et al., 2019). Nevertheless, there are no reports of *P. aeruginosa* infection nor a phage therapy model for murine mastitis. In this work, we successfully established a mouse model with mammary glands infected using *P. aeruginosa* strains isolated from cows with mastitis. In order to block adverse impact (reduced and variable disease induction) from suckling pups, we performed ‘forced weaning’ (removal of pups from the lactating female) before the time of mastitis induction (Ingman et al., 2015). However, prolonged forced weaning resulted in rapid accumulation of milk in the mammary gland causing some complication (Nazemi et al., 2014), which convoluted the analysis of mastitis induction. Therefore, consistent with most studies, we completed the experiment within 48 h following mastitis induction and forced weaning (Gonen et al., 2007; Breyne et al., 2014). Importantly, phage PAJD-1 exhibited the same satisfactory curative effect as antibiotics against mastitis infection. Moreover, no adverse effects were observed due to phage treatment in this study.

Compared with phages, the application of lysins, which are derived from phages, has been widely studied in recent years (Wang et al., 2018; Raz et al., 2019); in particular, lysins of phages that overcome Gram-positive pathogens infection (Rios et al., 2016; Wang et al., 2018). However, lysins were not initially recommended against Gram-negative pathogens because their impermeable outer membrane blocked lysin contact with peptidoglycans, which is the target of lysins (Gutierrez and Briers, 2020). Phage PAJD-1 had a strong bactericidal effect against a broad spectrum of bacteria, suggesting that the lysin encoded by the phage had a strong bactericidal effect. Therefore, the PlyPAJD-1 (lysin of PAJD-1) was expressed and purified. However, PlyPAJD-1 could not penetrate the outer membrane directly to kill bacteria, but it showed synergetic bactericidal efficacy when combined with EDTA, which disrupts the outer membrane by removing stabilizing cations and facilitating bacterial lysis by PlyPAJD-1 (Yang et al., 2018). At present, many studies have proposed different methods to transform lysin to effectively kill Gram-negative strains, including combining lysins with outer membrane permeabilizers (Oliveira et al., 2016), protein engineering (Wang et al., 2017; Yan et al., 2017) and formulating with nanocarriers (Ciepluch et al., 2019), which is the focus of our future research.

## Conclusion

In conclusion, the results presented herein provide insight into a lytic phage PAJD-1, which exhibited a wide host range, and strong lytic activity and stability under various conditions. Clearly, our animal experiments demonstrated that phage PAJD-1 can protect mice from mastitis infection by MDR *P. aeruginosa*. Thus, phage PAJD-1 may be an alternative antimicrobial agent in the clinic.

## Data Availability Statement

Publicly available datasets were analyzed in this study. This data can be found here: GenBank accession number: MW835180.

## Ethics Statement

The animal study was reviewed and approved by Ethical Committee for Animal Experiments of Shanghai Jiao Tong University.

## Author Contributions

YY, JS, and ZW designed the experiments. ZW, YX, and YG performed the experiments and collected the data. ZW, MG, YL, XZ, and YC collected and analyzed the data. JS, JM, and HW performed critical revision of the article. ZW wrote the manuscript. All authors contributed to the article and approved the submitted version.

## Funding

This study was funded by the Shanghai Agriculture Applied Technology Development Program, China (T20180205), the National Natural Science Foundation of China (31772744, 32072822, and 31902237), and the Science and Technology Commission of Shanghai Municipality (18391901900).

## Conflict of Interest

The authors declare that the research was conducted in the absence of any commercial or financial relationships that could be construed as a potential conflict of interest.
