# Supplementary material for: Phage vB_PaeS-PAJD-1 Rescues Murine Mastitis Infected With Multidrug-Resistant Pseudomonas aeruginosa
Source: Front Cell Infect Microbiol. 2021 Jun 11;11:689770. doi: 10.3389/fcimb.2021.689770 (PMC8226249; doi:10.3389/fcimb.2021.689770)

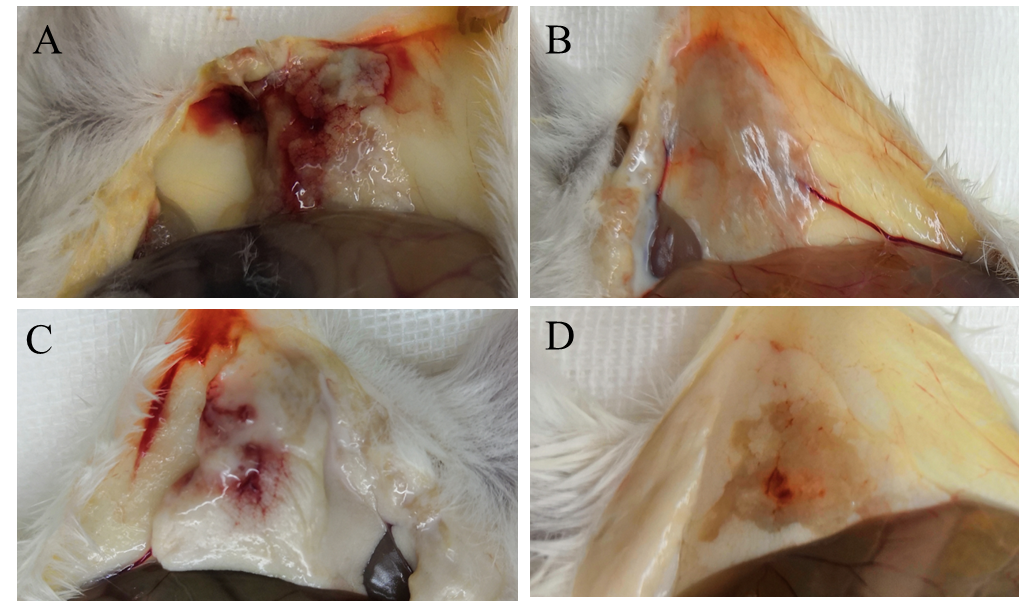


**Figure S1.** PAJD-1 reduced *P. aeruginosa*-induced mammary gland lesions of mice. After 24 h, the mammary glands of mice were photographed. Mice were infected with *P. aeruginosa* PAmas5 strains and treated with (C) PAJD-1 and (D) ceftiofur sodium. Mice were treated with PBS after infection as (A) a medium-treated group. (B) An uninfected mouse served as a positive control.

Table S1 Identification of *P. aeruginosa* PAmas-1 by the VITEK 2 system


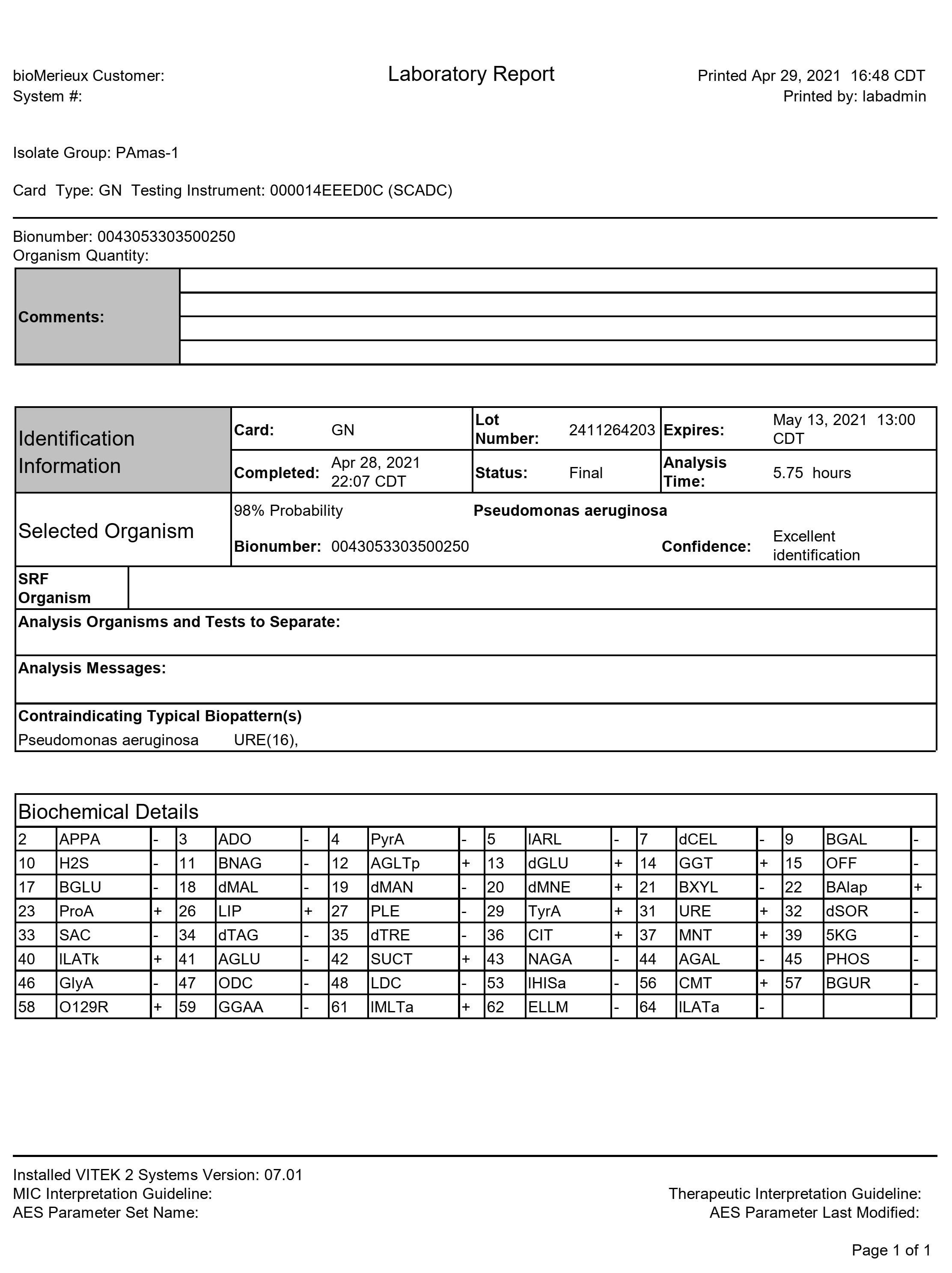


Table S2 Identification of *P. aeruginosa* PAmas-2 by the VITEK 2 system


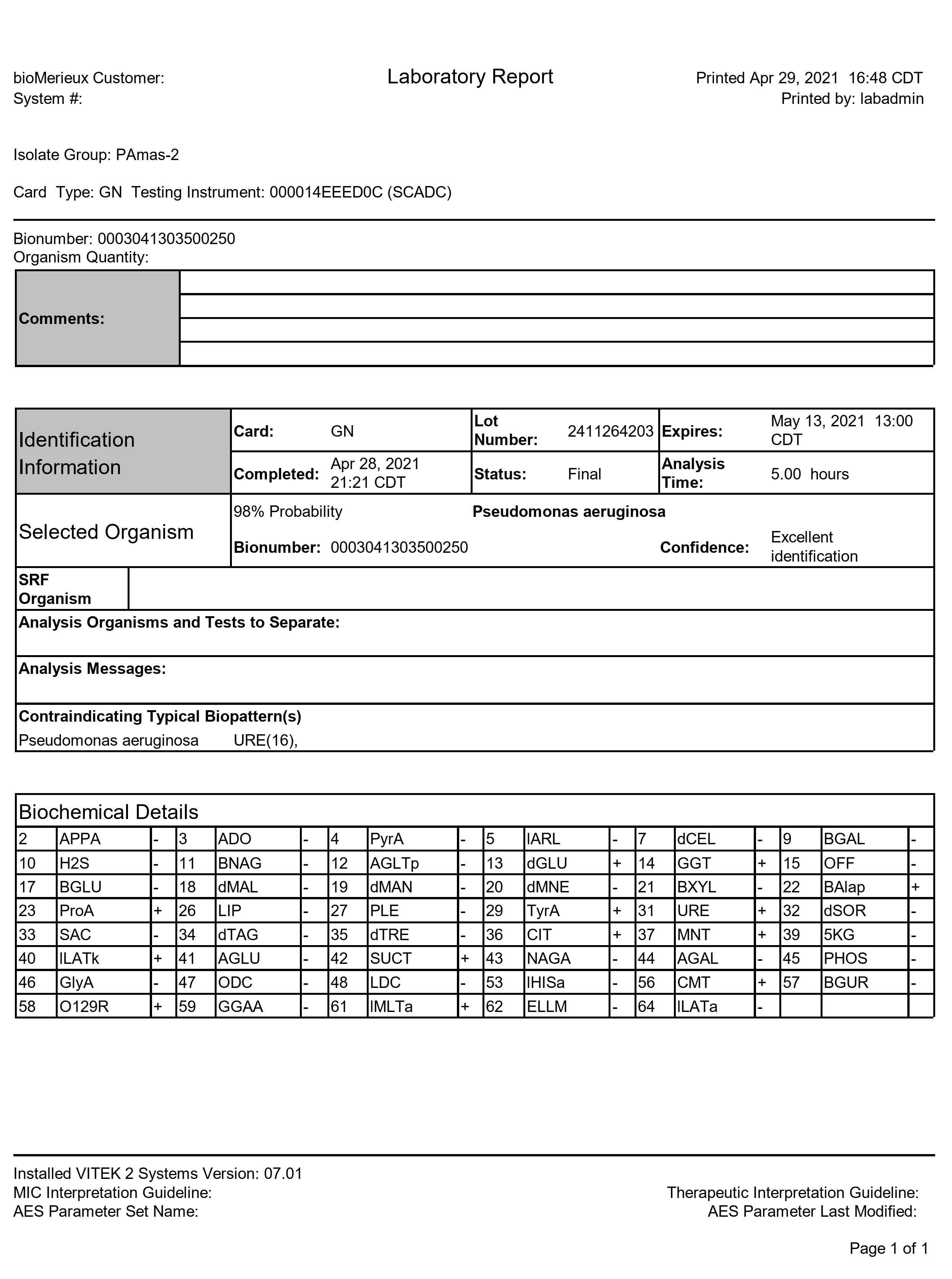


Table S3 Identification of *P. aeruginosa* PAmas-3 by the VITEK 2 system


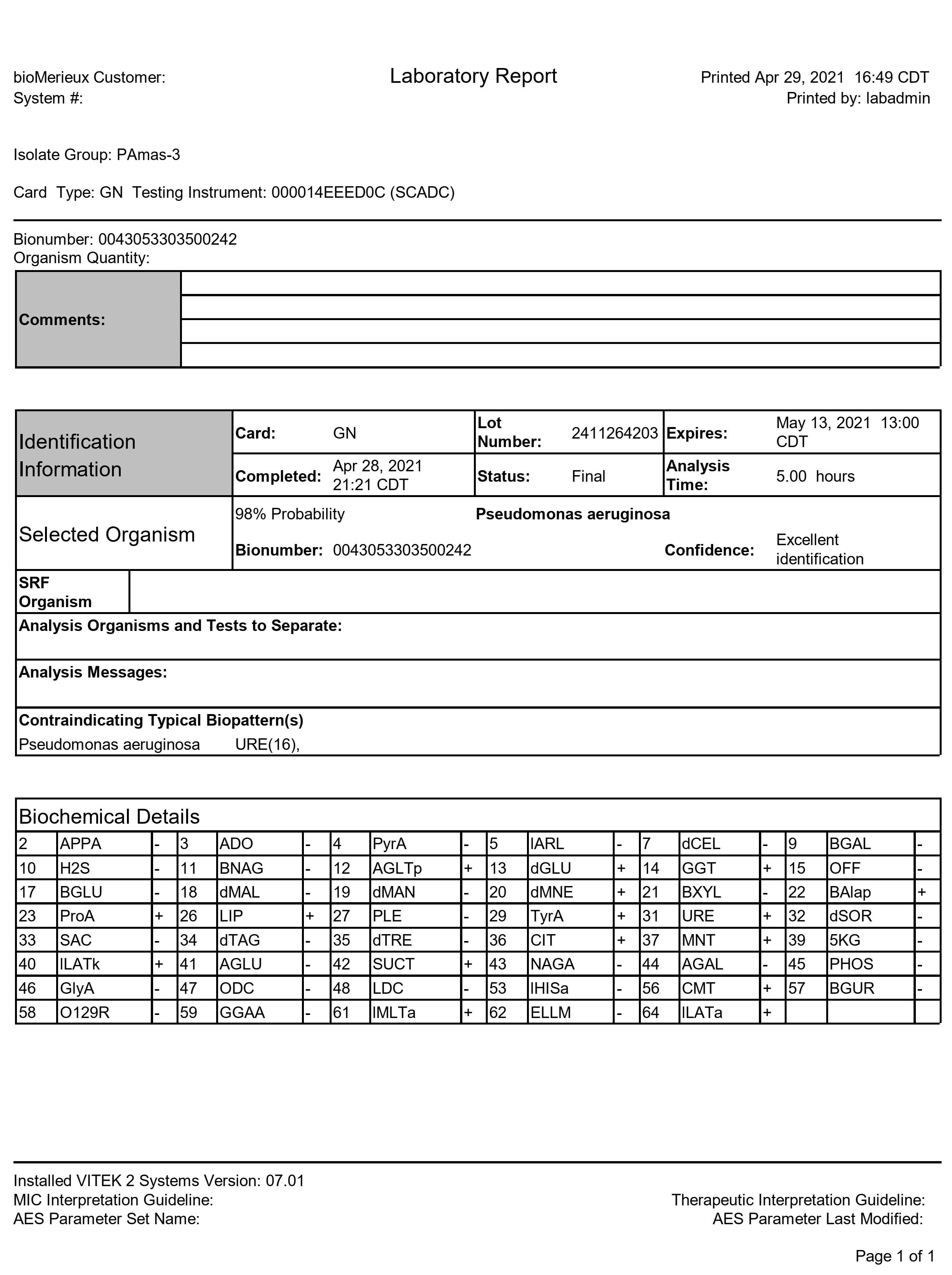


Table S4 Identification of *P. aeruginosa* PAmas-4 by the VITEK 2 system


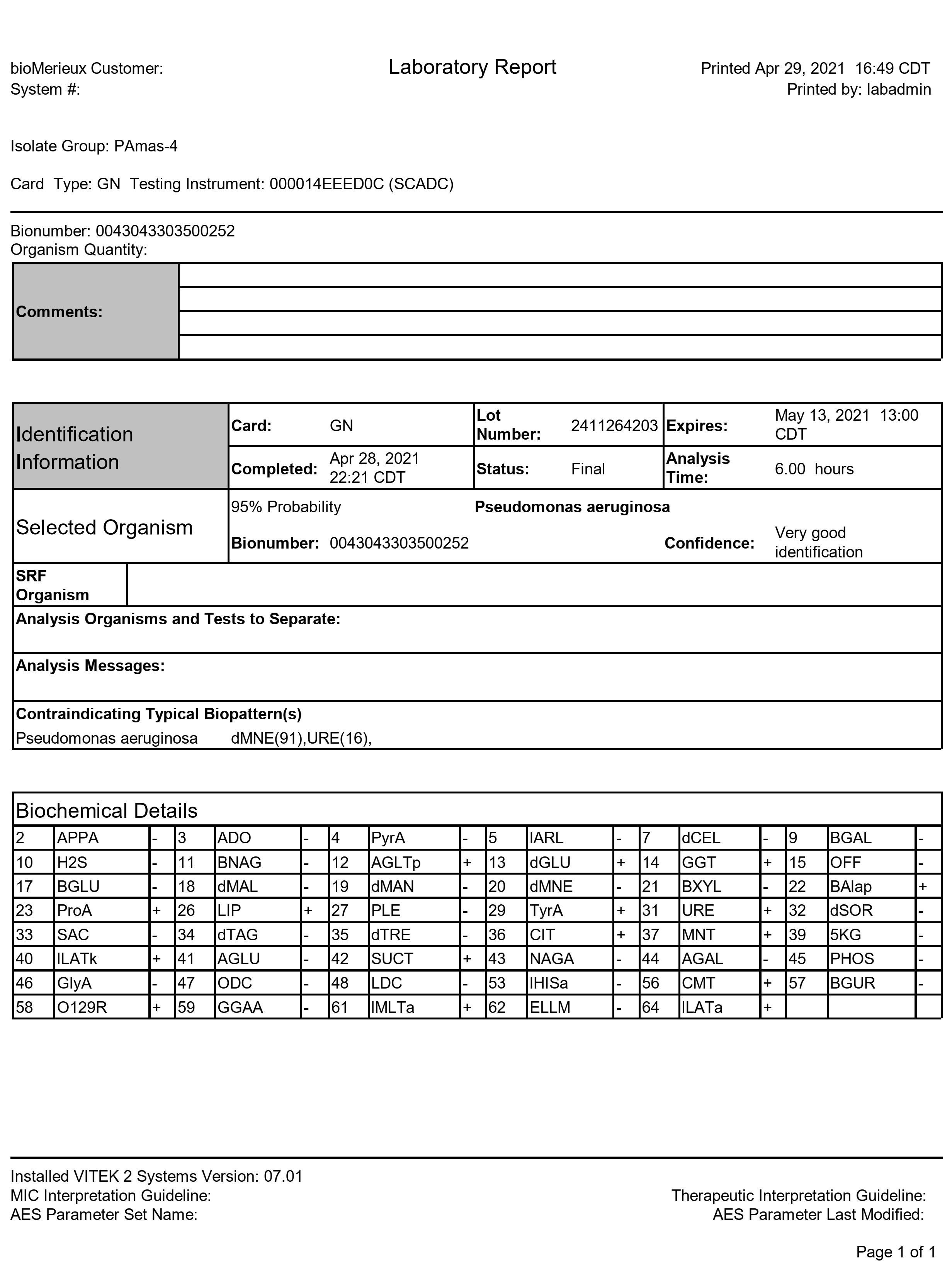


Table S5 Identification of *P. aeruginosa* PAmas-5 by the VITEK 2 system


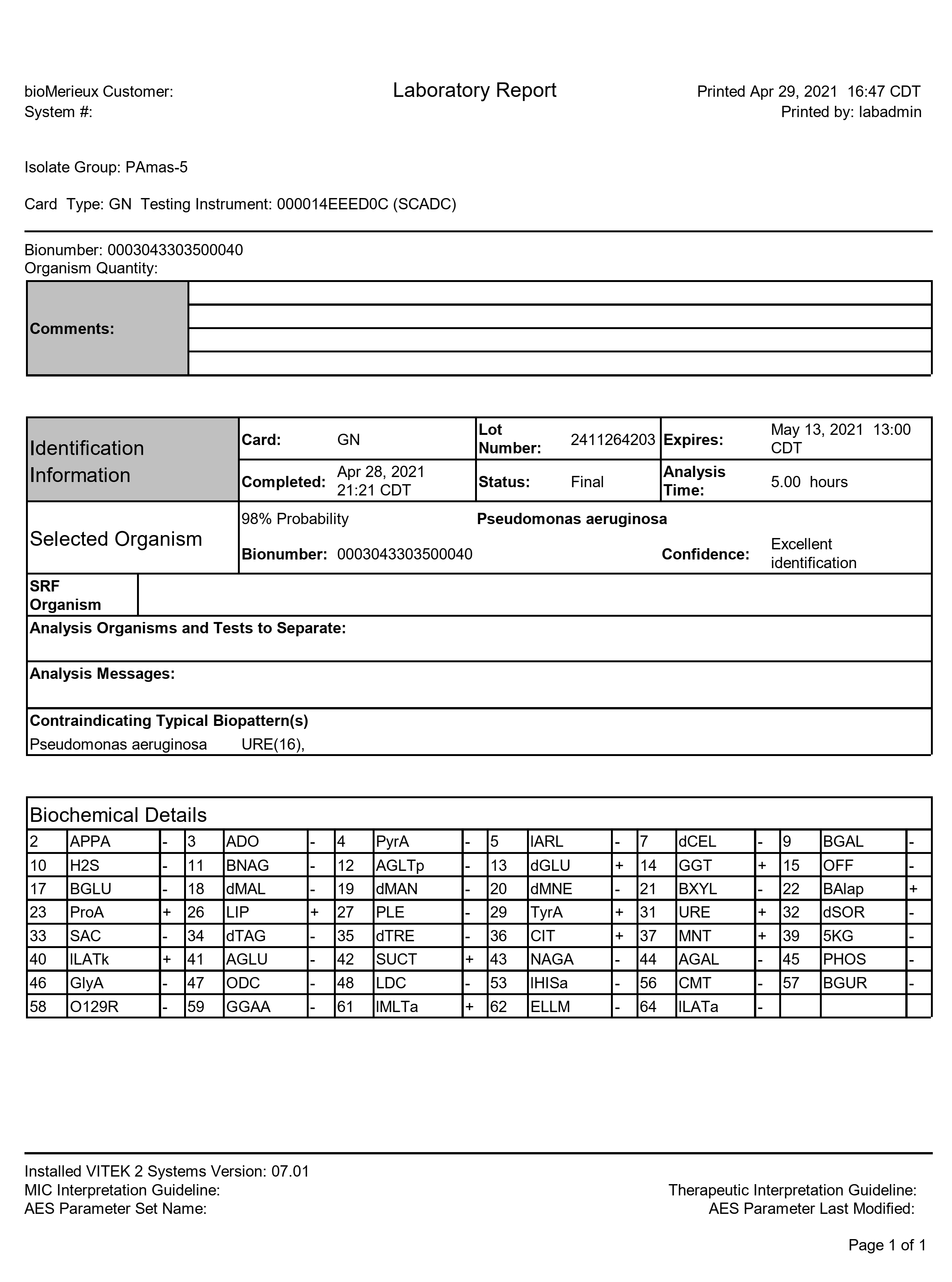

Supplement: Supplementary file 1 [file Table_1.docx]
